# Supplementary material for: Monitoring of cardiovascular physiology augmented by a patient-specific biomechanical model during general anesthesia. A proof of concept study
Source: PLoS One. 2020 May 14;15(5):e0232830. doi: 10.1371/journal.pone.0232830 (PMC7224549; doi:10.1371/journal.pone.0232830)
Supplement: Attachment — Submitted filename: PONE_rebuttal_letter_and_answers_to_reviewers.pdf [file pone.0232830.s002.pdf]

April 21, 2020

Dear Dr Ehab Farag,

We are glad to submit the revised version of our paper **"Monitoring of cardiovascular physiology augmented by a patient-specific biomechanical model during general anesthesia. A proof of concept study",** in which we addressed all the comments listed in the PLOS ONE decision email.

We would like to thank you and the reviewers for the feedback given to our manuscript. We are very pleased by the positive comments of reviewers, which we used to ameliorate the manuscript. In this rebuttal letter we are providing more details about the modifications in line with the editor's end reviewer's comments.

We hope you will take the revised version of our paper into consideration for publishing.

With kind regards,

R. Chabiniok et al

1. Please ensure that your manuscript meets PLOS ONE's style requirements, including those for file naming.

We revised journal requirements and adapted the style and file naming accordingly.

2. Please note that PLOS ONE has specific guidelines on software sharing (https://journals.plos.org/plosone/s/materials-and-software-sharing#loc-sharing-software) for manuscripts whose main purpose is the description of a new software or software package. In this case, new software must conform to the Open Source Definition (https://opensource.org/docs/osd) and be deposited in an open software archive. Please see https://journals.plos.org/plosone/s/materials-and-software-sharing#loc-depositing-softwarefor more information on depositing your CardiacLab library, if you have not already done so.

The purpose of the article is not to describe a new software or software package. The model used in this paper is fully described within the manuscript and the cited material (in particular [Caruel et al, BMMB2014], [Sainte-Marie_C&S2006], and [Chapelle et al, INT J MULTISCALE COM2012]). CardiacLab is just an internal code and we therefore retract this internal name from the manuscript. The modified closing paragraph of the Methods therefore reads:

"*The simulations were performed using an in-house implementation of the model in MATLAB (The MathWorks Inc, Natick, Massachusetts).*"

3. We note that you have stated that you will provide repository information for your data at acceptance. Should your manuscript be accepted for publication, we will hold it until you provide the relevant accession numbers or DOIs necessary to access your data. If you wish to make changes to your Data Availability statement, please describe these changes in your cover letter and we will update your Data Availability statement to reflect the information you provide.

All data are available within the Supplementary material S1_file.txt. The Data Availability statement is therefore reads:

"All relevant data are within the manuscript and its Supporting Information files."

4. We note that you have a patent relating to material pertinent to this article. Please provide an amended statement of Competing Interests to declare this patent (with details including name and number), along with any other relevant declarations relating to employment, consultancy, patents, products in development or modified products etc. Please confirm that this does not alter your adherence to all PLOS ONE policies on sharing data and materials, as detailed online in our guide for authors http://journals.plos.org/plosone/s/competing-interestsby including the following statement: "This

does not alter our adherence to PLOS ONE policies on sharing data and materials." If there are restrictions on sharing of data and/or materials, please state these. Please note that we cannot proceed with consideration of your article until this information has been declared.

We would like to amend our competing interest statement

"A.L.G., F.V, D.C. and R.C. are co-owners of the patent entitled "Dispositif cardiaque" (number 1758006, 2017). A research license agreement is currently ongoing between the Anesthesiology and intensive care department of Lariboisi\`ere hospital, Paris, France and Deltex Medical, Chichester, UK. "

to:

"*I have read the journal's policy and the authors of this manuscript have the following competing interests: A.L.G., F.V, D.C. and R.C. are co-owners of the patent entitled "Dispositif cardiaque" (number 1758006, 2017). A research license agreement is currently ongoing between the Anesthesiology and intensive care department of Lariboisi\`ere hospital, Paris, France and Deltex Medical, Chichester, UK.* This does not alter our adherence to PLOS ONE policies on sharing data and materials."

5. Review Comments to the Author

Please use the space provided to explain your answers to the questions above. You may also include additional comments for the author, including concerns about dual publication, research ethics, or publication ethics. (Please upload your review as an attachment if it exceeds 20,000 characters)

Reviewer #1: Very well done and interesting study.As the authors describe,paves the way for future studies based on their modeling.Good statistical analysis.Diagrams are well done too.No further changes recommended.

We are grateful for such a positive feedback.

Reviewer #2: This interesting and innovative study sought to improve the state-of-the-art of hemodynamic monitoring by developing a method for constructing patient-specific bio-mechanical models of the heart and circulation. Such models may offer future clinicians a step forward in the physiological monitoring and pharmacological management of ICU patients and patients undergoing general anesthesia.

The science behind the author's efforts is quite complex, but adequately described.

However, a major concern I have is the confusing use of English language throughout the manuscript. Two examples are the following:

around 230,000,000 general anesthesia (GA) are performed each year worldwide

and

titration of saline isochloride by 250 ml (Do you mean Normal Saline?)

The manuscript was proofread in detail and English ameliorated (the changes are indicated in the manuscript with track changes). In particular:

"around 230,000,000 general anesthesia (GA) are performed each year ..." was replaced by
" *around 230 million major surgical procedures under general anesthesia (GA) are performed each year ....*"

And

" titration of saline isochloride by 250 ml step" was replaced by
"*titration of saline solution by 250 ml steps "*

The authors note that the study "simulations were performed using the CardiacLab library", an in-house developed MATLAB model. Is this MATLAB code available for download by other investigators?

As in Point 2:

The purpose of the article is not to describe a new software or software package. The model used in this paper is fully described within the manuscript and the cited material (in particular [Caruel et al, BMMB2014], [Sainte-Marie_C&S2006], and [Chapelle et al, INT J MULTISCALE COM2012]). CardiacLab is just an internal code and we therefore retract this internal name from the manuscript. The modified closing paragraph of the Methods therefore reads:

"*The simulations were performed using an in-house implementation of the model in MATLAB (The MathWorks Inc, Natick, Massachusetts).*"

One final suggestion I have is to make more explicit the limitations of the present study in a separate section."

In the revised manuscript, we extended the limitations and presented them in a separate section.

PONE-D-20-06385
Monitoring of cardiovascular physiology augmented by a patient-specific biomechanical model during general anesthesia. A proof of concept study.
PLOS ONE

Dear Dr. Radomír Chabiniok

Thank you for submitting your manuscript to PLOS ONE. After careful consideration, we feel that it has merit but does not fully meet PLOS ONE's publication criteria as it currently stands. Therefore, we invite you to submit a revised version of the manuscript that addresses the points raised during the review process.

I would appreciate if you response to the reviewers' comments

We would appreciate receiving your revised manuscript by May 17 2020 11:59PM. When you are ready to submit your revision, log on to https://www.editorialmanager.com/pone/ and select the 'Submissions Needing Revision' folder to locate your manuscript file.

If you would like to make changes to your financial disclosure, please include your updated statement in your cover letter.

To enhance the reproducibility of your results, we recommend that if applicable you deposit your laboratory protocols in protocols.io, where a protocol can be assigned its own identifier (DOI) such that it can be cited independently in the future. For instructions see: http://journals.plos.org/plosone/s/submission-guidelines#loc-laboratory-protocols

Please include the following items when submitting your revised manuscript:

- A rebuttal letter that responds to each point raised by the academic editor and reviewer(s). This letter should be uploaded as separate file and labeled 'Response to Reviewers'.
- A marked-up copy of your manuscript that highlights changes made to the original version. This file should be uploaded as separate file and labeled 'Revised Manuscript with Track Changes'.
- An unmarked version of your revised paper without tracked changes. This file should be uploaded as separate file and labeled 'Manuscript'.

Please note while forming your response, if your article is accepted, you may have the opportunity to make the peer review history publicly available. The record will include editor decision letters (with reviews) and your responses to reviewer comments. If eligible, we will contact you to opt in or out.

We look forward to receiving your revised manuscript.

Kind regards,

Ehab Farag, MD FRCA FASA
Academic Editor
PLOS ONE

Journal requirements:

When submitting your revision, we need you to address these additional requirements:

1. Please ensure that your manuscript meets PLOS ONE's style requirements, including those for file naming. The PLOS ONE style templates can be found at http://www.plosone.org/attachments/PLOSOne_formatting_sample_main_body.pdf and http://www.plosone.org/attachments/PLOSOne_formatting_sample_title_authors_affiliations.pdf

2. Please note that PLOS ONE has specific guidelines on software sharing (https://journals.plos.org/plosone/s/materials-and-software-sharing#loc-sharing-software) for manuscripts whose main purpose is the description of a new software or software package. In this case, new software must conform to the Open Source Definition (https://opensource.org/docs/osd) and be deposited in an open software archive. Please see https://journals.plos.org/plosone/s/materials-and-software-sharing#loc-depositing-softwarefor more information on depositing your CardiacLab library, if you have not already done so.

3. We note that you have stated that you will provide repository information for your data at acceptance. Should your manuscript be accepted for publication, we will hold it until you provide the relevant accession numbers or DOIs necessary to access your data. If you wish to make changes to your Data Availability statement, please describe these changes in your cover letter and we will update your Data Availability statement to reflect the information you provide.

4. We note that you have a patent relating to material pertinent to this article. Please provide an amended statement of Competing Interests to declare this patent (with details including name and number), along with any other relevant declarations relating to employment, consultancy, patents, products in development or modified products etc. Please confirm that this does not alter your adherence to all PLOS ONE policies on sharing data and materials, as detailed online in our guide for authors http://journals.plos.org/plosone/s/competing-interestsby including the following statement: "This does not alter our adherence to PLOS ONE policies on sharing data and materials." If there are restrictions on sharing of data and/or materials, please state these. Please note that we cannot proceed with consideration of your article until this information has been declared.

This information should be included in your cover letter; we will change the online submission form on your behalf.

Please know it is PLOS ONE policy for corresponding authors to declare, on behalf of all authors, all potential competing interests for the purposes of transparency. PLOS defines a competing interest as anything that interferes with, or could reasonably be perceived as interfering with, the full and objective presentation, peer review, editorial decision-making, or publication of research or non-research articles submitted to one of the journals. Competing interests can be financial or non-financial, professional, or personal. Competing interests can arise in relationship to an organization or another person. Please follow this link to our website for more details on competing interests: http://journals.plos.org/plosone/s/competing-interests

[Note: HTML markup is below. Please do not edit.]

Reviewers' comments:

Reviewer's Responses to Questions

Comments to the Author

1. Is the manuscript technically sound, and do the data support the conclusions?

The manuscript must describe a technically sound piece of scientific research with data that supports the conclusions. Experiments must have been conducted rigorously, with appropriate controls, replication, and sample sizes. The conclusions must be drawn appropriately based on the data presented.

Reviewer #1: Yes

Reviewer #2: Yes

---

2. Has the statistical analysis been performed appropriately and rigorously?

Reviewer #1: Yes

Reviewer #2: I Don't Know

---

3. Have the authors made all data underlying the findings in their manuscript fully available?

The PLOS Data policy requires authors to make all data underlying the findings described in their manuscript fully available without restriction, with rare exception (please refer to the Data Availability Statement in the manuscript PDF file). The data should be provided as part of the manuscript or its supporting information, or deposited to a public repository. For example, in addition to summary statistics, the data points behind means, medians and variance measures should be available. If there are restrictions on publicly sharing data—e.g. participant privacy or use of data from a third party—those must be specified.

Reviewer #1: Yes

Reviewer #2: Yes

---

4. Is the manuscript presented in an intelligible fashion and written in standard English?

PLOS ONE does not copyedit accepted manuscripts, so the language in submitted articles must be clear, correct, and unambiguous. Any typographical or grammatical errors should be corrected at revision, so please note any specific errors here.

Reviewer #1: Yes

Reviewer #2: Yes

5. Review Comments to the Author

Please use the space provided to explain your answers to the questions above. You may also include additional comments for the author, including concerns about dual publication, research ethics, or publication ethics. (Please upload your review as an attachment if it exceeds 20,000 characters)

Reviewer #1: Very well done and interesting study.As the authors describe,paves the way for future studies based on their modeling.Good statistical analysis.Diagrams are well done too.No further changes recommended.

Reviewer #2: This interesting and innovative study sought to improve the state-of-the-art of hemodynamic monitoring by developing a method for constructing patient-specific bio-mechanical models of the heart and circulation. Such models may offer future clinicians a step forward in the physiological monitoring and pharmacological management of ICU patients and patients undergoing general anesthesia.

The science behind the author's efforts is quite complex, but adequately described.

However, a major concern I have is the confusing use of English language throughout the manuscript. Two examples are the following:

around 230,000,000 general anesthesia (GA) are performed each year worldwide

and

titration of saline isochloride by 250 ml (Do you mean Normal Saline?)

The authors note that the study "simulations were performed using the CardiacLab library", an in-house developed MATLAB model. Is this MATLAB code available for download by other investigators?

One final suggestion I have is to make more explicit the limitations of the present study in a separate section.

---

6. PLOS authors have the option to publish the peer review history of their article (what does this mean?). If published, this will include your full peer review and any attached files.

If you choose "no", your identity will remain anonymous but your review may still be made public.

Do you want your identity to be public for this peer review? For information about this choice, including consent withdrawal, please see our Privacy Policy.

Reviewer #1: No

Reviewer #2: No

[NOTE: If reviewer comments were submitted as an attachment file, they will be attached to this email and accessible via the submission site. Please log into your account, locate the manuscript record, and check for the action link "View Attachments". If this link does not appear, there are no attachment files to be viewed.]

While revising your submission, please upload your figure files to the Preflight Analysis and Conversion Engine (PACE) digital diagnostic tool, https://pacev2.apexcovantage.com/. PACE helps ensure that figures meet PLOS requirements. To use PACE, you must first register as a user. Registration is free. Then, login and navigate to the UPLOAD tab, where you will find detailed instructions on how to use the tool. If you encounter any issues or have any questions when using PACE, please email us at figures@plos.org. Please note that Supporting Information files do not need this step.
